# Tuberous-sclerosis complex-related cell signaling in the pathogenesis of lung cancer

**DOI:** 10.1186/1746-1596-9-48

**Published:** 2014-03-04

**Authors:** Angela Fuchs, Katharina König, Lukas C Heukamp, Jana Fassunke, Jutta Kirfel, Sebastian Huss, Albert J Becker, Reinhard Büttner, Michael Majores

**Affiliations:** 1 Department of Cardiology, Angiology and Pneumology, University of Bonn Medical School, Sigmund-Freud-Strasse 25, Bonn 53127, Germany; 2 Institute of Pathology, University of Cologne, Kerpener Strasse 62, Köln 50937, Germany; 3 Institute of Pathology, University of Bonn Medical School, Sigmund-Freud-Strasse 25, Bonn 53127, Germany; 4 Institute of Neuropathology, University of Bonn Medical School, Sigmund-Freud-Strasse 25, Bonn 53127, Germany; 5 Gerhard-Domagk-Institute of Pathology, University Hospital Münster, Albert-Schweitzer-Campus 1, Münster 48149, Germany

**Keywords:** Tuberous sclerosis complex, TSC, EGFR, Hamartin, Tuberin, Lung cancer

## Abstract

**Background:**

Hamartin (TSC1) and tuberin (TSC2), encoded by the tuberous sclerosis complex (*TSC*) genes, form a tumor-suppressor heterodimer which is implicated in PI3K-Akt signaling and acts as a functional inhibitor of the mammalian target of rapamycin (mTOR). Dysregulation of mTOR has been assigned to carcinogenesis and thus may be involved in cancer development. We have addressed the role of hamartin, phospho-tuberin (p-TSC2) and phospho-mTOR (p-mTOR) in a series of non-small cell lung cancer (NSCLC) and small cell lung cancer (SCLC) samples.

**Methods:**

We collected 166 NSCLC and SCLC samples for immunohistochemical studies and performed western blot analyses in NSCLC and SCLC cell lines as well as comparative analyses with EGFR phosphorylation and downstream effectors.

**Results:**

In cell lines we found an inverse correlation between hamartin and p-mTOR expression. In surgical specimens cytoplasmic hamartin expression was observed in more than 50% of adenocarcinoma (AC) and squamous cell carcinoma (SCC) compared to 14% of SCLC. P-mTOR and p-TSC2 staining was found in a minority of cases.

There was a significant correlation between p-EGFR Tyr-1068, p-EGFR Tyr-992 and hamartin, and also between p-mTOR and p-EGFR Tyr-1173 in AC. In SCC an inverse correlation between hamartin and p-EGFR Tyr-992 was detected. Phosphorylation of TSC2 was associated with expression of MAP-Kinase. Hamartin, p-TSC2 and p-mTOR expression was not dependant of the EGFR mutation status. Hamartin expression is associated with poorer survival in SCC and SCLC.

**Conclusions:**

Our findings confirm the inhibitory role of the tuberous sclerosis complex for mTOR activation in lung cancer cell lines. These results reveal hamartin expression in a substantial subset of NSCLC and SCLC specimens, which may be due to EGFR signaling but is not dependant on EGFR mutations. Our data provide evidence for a functional role of the tuberous sclerosis complex in lung cancer.

**Virtual slides:**

The virtual slide(s) for this article can be found here: http://www.diagnosticpathology.diagnomx.eu/vs/9274845161175223.

## Background

Lung cancer is one of the most common cancer diseases and a major tumor-related cause of death in western industrialized countries, accounting for more than 1 million new cases and deaths each year (12.6% of all new cancers, 17.8% of cancer related deaths). According to the WHO classification of 2004 malignant epithelial lung tumors are classified into main subsets based on histomorphologic and immunohistochemical features [1]. These subsets comprise squamous cell, small cell, large cell, adenosquamous, sarcomatoid carcinomas and adenocarcinomas comprising different subtypes [2,3]. Despite major efforts in standardized diagnostic and therapeutic procedures, patients’ overall survival remains poor, i.e. complete remission and long-term survival is only rarely achieved.

**Figure 1:**
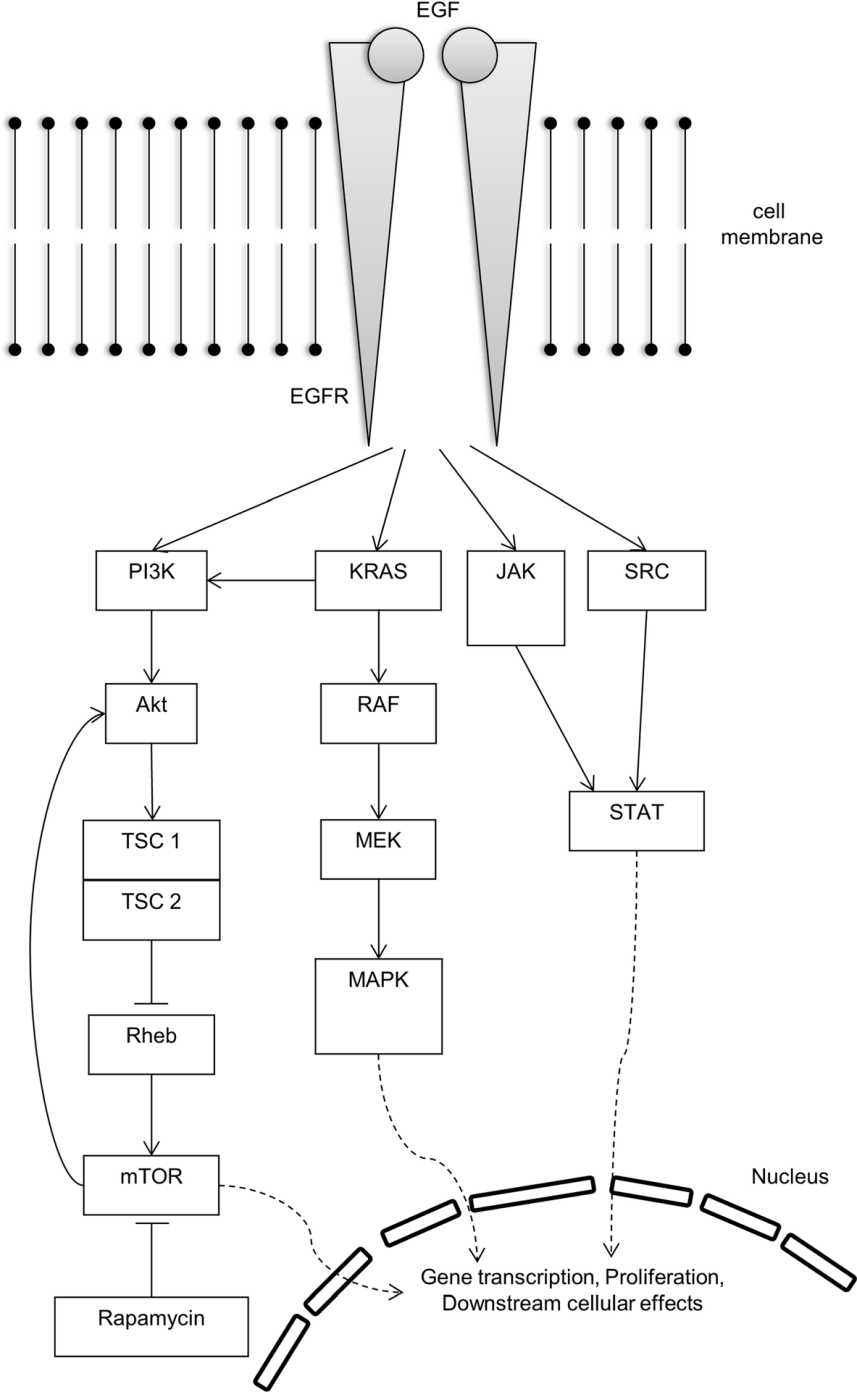
**EGFR associated signaling pathways in lung cancer.** Phosphorylation, i.e. activation of the Epidermal Growth Factor Receptor (EGFR) results in various downstream signaling pathways including the JAK-STAT-signaling, KRAS, MAPK and the phosphatidylinositol 3-kinase (PI3K) related pathway. Extrinsic binding of the Endothelian Growth Factor (EGF) to the corresponding Endothelian Growth Factor Receptor (EGFR) leads to phosphorylation of EGFR and subsequent activation of phosphatidylinositol 3-kinase (PI3K). PI3K signaling results in an activation of Akt and TSC1/TSC2, diminishing its inhibitory effect on Rheb (Ras homolog enriched in brain), a major activator of mTOR signaling. Disruption of the TSC-complex leads to activation (disinhibition) of mTOR and may play a putative pathogenic role in lung cancer pathogenesis. *Arrows represent activation; bars represent inhibitory effects.*

A better understanding of the molecular mechanisms of carcinogenesis and disease progression is essential for the development of targeted therapies [4]. Growing evidence supports the pathogenic role of abnormal EGFR-related cell signaling, thereby affecting various downstream signaling cascades (Figure 1). Activation of the EGFR pathway mediated by activating mutations in its constituents is a key driver in adenocarcinomas of the lung, mediating important carcinogenic properties such as cell-cycle progression, apoptosis, angiogenesis and metastasis [5]. Distinct activating in EGFR and activation of associated signaling pathways is a well-established finding in upto 20% of adenocarcinoma located in the lung [6,7] and tyrosine-kinase inhibitors (TKI) directed against the EGFR have entered clinical practice. EGFR-related downstream effects are mediated by the phosphatidylinositol 3-kinase (PI3K) - Akt signaling cascade, which promotes tumor cell growth and inhibition of apoptosis by activation of mTOR [7-9]. An exact characterization of EGFR mutations has therefore become essential to determine therapeutic options and assess potential therapy failure due to secondary resistance to TKI-therapy; e.g. recent mutation analysis revealed a new activating mutation in Exon 19 in the EGFR gene in a liver metastasis of a primary lung adenocarcinoma with therapeutical potential [10]. Furthermore there are large efforts and promising results regarding optimization of immunohistochemical markers as prescreening tests to detect EGFR mutations in potential TKI candidates [11].

The present study is focusing on a central regulator of the EGFR-dependant PI3K - mTOR pathway, i.e. the tuberous sclerosis tumor suppressor complex (TSC). The TSC complex is constituted by a heterodimer of hamartin and tuberin, encoded by the *TSC1* and *TSC2* genes. Germline mutations of the *TSC1* and *TSC2* genes cause the familial syndrome of tuberous sclerosis complex [12]. Those patients suffer from hamartomas and tumors in various tissues such as kidney angiomyolipoma, cardiac rhabdomyoma, subependymal giant cell astrocytoma (SEGA) and increased risk for renal cancer [13]. TSC acts via the GAP protein Rheb (Ras homolog enhanced in brain) and thereby leads to an inhibition of mTOR [14]. Vice versa, disruption of the TSC tumor suppressor complex results in an upregulation of mTOR. Moreover, mTOR signaling may be interfered by Rapamycin, a negative regulator of mTOR [15]. A pathogenic role of the TSC tumor-suppressor complex has been described in diverse sporadic malignant neoplasms, such as sporadic bladder cancer, breast cancer, ovarian carcinoma and gall bladder carcinoma [16,17]. In lung cancer, only sparse data concerning a putative pathogenic role of the TSC complex are available. A loss of heterozygosis (LOH) of the TSC1-locus on chromosome 9q34 was observed in AC and precursor lesions, i.e. atypical adenomatous hyperplasia [18]. Furthermore, TSC1 mutations and polymorphisms, but no truncating mutations were found in AC specimens [19]. Another study reported LOH for hamartin or TSC2 in 22% of 86 specimens, but none of the 80 lung cancer lines studied showed lack of expression or complete loss of either hamartin or TSC2 [20]. This is the first comprehensive immunohistochemical and clinicopathological study of the Tuberous-sclerosis complex-related cell signaling in the pathogenesis of lung cancer.

## Methods

### Patients/specimens

In total, 166 patient samples were included in the study and chosen from the archival files of the Institute of Pathology, University Bonn Medical School. Patients suffered from primary malignant tumors of the lung and were subjected to surgical resection or diagnostic sampling between June 1995 and December 2006. In each case, the primary pulmonary origin of the neoplasms was affirmed by a panel of immunohistochemical analyses by experienced pathologists. The male: female ratio (109: 57), mean age at diagnosis (64.2 years; SD +/− 9.8 years; range 33 – 86 years) and follow-up characteristics of our cohort were in accordance with the published epidemiologic distribution and clinical course.

Tumor samples derived from formalin-fixed, paraffin-embedded tissue. According to the current WHO classification (2004) these samples were classified in 92 AC, 31 SCC and 43 SCLC. For NSCLC specimens we created tissue microarrays (TMAs) in paraffin embedded tissue. NSCLC and unaffected tissue specimens were analyzed using TMAs comprising 3 tissue samples of 1.6 mm diameter of each case. To verify the representativeness of TMA-immunohistochemistry, whole block sections of 44 cases randomly chosen among the malignant tissue series were stained in parallel. Only one of 44 cases revealed to be false-negative and vice-versa, 9 of 10 positive samples in the whole paraffine block specimens were also found positive in the TMA specimens. Therefore were considered TMAs to be valid for immunohistochemical characterization of NSCLC specimens. Due to limited availability of resected specimens, SCLC were examined by staining whole sections of biopsied samples.

### Immunohistochemistry

The classification of tumors was based on hematoxylin and eosin staining, and ascertained by the use of primary antibodies for the detection of TTF-1 (monoclonal, diluted 1:50, DAKO, Hamburg, Germany), CD56 (monoclonal, diluted, 1:50, DAKO), CK5/6 (monoclonal, diluted 1:50, DAKO), and CK7 (monoclonal, diluted 1:500, DAKO), using standard protocols. For the present study, the following primary antibodies were used for immunohistochemical staining: P-mTOR (Ser2448) (polyclonal, diluted 1:100, Cell Signaling, Frankfurt, Germany), hamartin/TSC1 (1B2) (monoclonal, diluted 1:200, Cell Signaling) and p-tuberin/TSC2 (Thr1462) (5B12) (monoclonal, diluted 1:100, Cell Signaling). Antibody incubation was performed over night at 4°C after heat treatment of the slides (DakoCytomation Pascal pressure chamber, 3 minutes in 0.01 M sodium citrate buffer, ph 6.0). Negative control reactions replacing the primary specific antibody by nonspecific immunoglobulin were performed exemplarily to assure specific binding affinity. Endogenous peroxidase activity was quenched by incubation with DakoReal Peroxidase-Blocking Solution S2023 for 5 minutes. Blocking of nonspecific binding was performed with DakoReal Buffer Kit K5006, containing carrier protein, detergent and preservative. Biotinylated secondary antibody (incubation of 30 minutes at room temperature) and Strepavidin/HRP incubation was performed using the DakoReal Detection System. Sections were counterstained with hematoxylin. Additionally, we performed statistical comparisons with immunohistochemical data obtained in a recent project concerning signaling pathways in lung cancer pathogenesis (p-EGFR, Stat3, Akt) [21]. Immunostaining was analysed semiquantitatively in a four-tiered scoring approach, assigning TMA samples to 0) negative, 1) equivocal, 2) weak to moderate, and 3) strong. Statistical analysis was performed using a Fischer’s exact test.

### Cell culture

In this work 5 cell lines of SCLC and NSCLC were examined. All cell lines were cultured in an incubator at 37°C and 5% CO2 in 75 cm^2^ tissue culture flasks (CORNING, Kaiserslautern, Germany) containing 15 ml of sterile medium. The following cell lines were used: “NCI-H460”, a human large cell lung carcinoma cell line bearing a K-Ras G61 H mutation; “MBA 9812 16B13”, a squamous cell carcinoma cell line; “HCC827”, an adenocarcinoma cell line harboring an acquired mutation within the EGFR tyrosine kinase domain (E746-A750 deletion). SCLC cell lines: “GLC-2” and “GLC-8”. The cell lines NCI-H460, GLC-2 and MBA-9812-16B13 were routinely cultured in RPMI 1640 medium (Invitrogen, Karlsruhe, Germany) supplemented with 8% fetal bovine serum (Invitrogen), L-glutamine (GIBCO), 1 mmol Sodium-Pyruvate (Invitrogen), penicillin/streptomycin (PAA, Cölbe, Germany), and β-mercaptoethanol (Merck). GLC-8 and HCC827 were cultured in IMDM (PAA) supplemented with additions as above. Growth media were changed at least after 48–72 h.

### Western blot analyses

Protein isolation was performed by harvesting 5 × 10^6^ cells and centrifugation at 3000 rpm and 4°C for 3 minutes. The pellet was dissolved in 100 μl RIPA- Buffer (SIGMA-ALDRICH, Germany) and incubated on ice for 30 minutes. Centrifugation at 13000 rpm and 4°C for 15 minutes finally enabled to take the supernatant which contained the proteins. The extracted protein concentrations were measured according to the method of Bradford. Protein lysates from 50000 cells were supplemented with NuPage LDS Sample Buffer (4 ×) (Invitrogen), NuPage Sample Reducing Agent (10 ×) (Invitrogen), PBS (PAA) and denaturized at 95°C for 5 minutes. Proteins were loaded on NuPage 4-12% Bis-Tris Gel (1.5 mm × 15 well, Invitrogen), placed in Xcell Sure Lock Mini-Cell device (Invitrogen), filled with MOPS SDS Running buffer (Invitrogen) and separated at 170 V for 1 h30. Magic Mark XP Western Standard (Invitrogen) for hamartin/TSC1 and HiMark Pre-Stained High Molecular Weight Protein Standard (Invitrogen) for P-mTOR and P-tuberin/TSC2 were used to make protein sizes comparable. Proteins were transferred to a nitrocellulose membrane using Xcell II Blot Module (Invitrogen) filled with NuPage Transfer Buffer without methanol at 30 V for 1 h40. After blocking in 5% nonfat drymilk/TBST (Tris-buffered saline solution containing 0.1% Tween 20) for 1 hour at room temperature the membranes were incubated with a polyclonal rabbit primary anti-p-mTOR (Ser2448) and anti-p-tuberin /TSC2 (Thr1462) (5B12) antibody (Cell Signaling) as well as a monoclonal mouse anti-hamartin/TSC1 antibody (1B2) (Cell Signaling) in 5% nonfat drymilk/TBST at a dilution of 1:1000 over night at 4°C in 5% BSA/TBST. Next to that they were washed 3 times for 10 min each and incubated with HRP-Goat Anti-Rabbit IgG secondary antibody (Zymed, Invitrogen) for p-mTOR (Ser2448) and p-tuberin /TSC2 (Thr1462) (5B12) at a dilution of 1:4000 in 5% nonfat drymilk/TBST for 1 hour at room temperature meanwhile hamartin/TSC1 (1B2) was incubated with HRP-Goat Anti-Mouse secondary antibody (SIGMA-ALDRICH) at a dilution of 1:2000 in 5% nonfat drymilk/TBST. Immunoreactive proteins were visualized with 0.125 ml/cm2 ECL Western blotting detection reagents and analysis system (GE Healthcare, Amersham).

### DNA extraction, polymerase chain reaction (PCR) and DNA sequencing

DNA was isolated from the cultured cell lines (n = 5) and 1 blood sample arising from a healthy person as additional control. DNA was isolated and purified using a QIAamp DNA Mini Kit (QIAGEN, Germany) according to the manufacturer’s instructions for cultured cells or rather whole blood. Amplification was performed in a 25 μl reaction mixture containing 20 ng DNA, 0.2 mM of each dNTP, 0.2 μl Taq DNA polymerase (Invitrogen, 5 U/μl), 2.5 μl 10 × Buffer, 1 μl of 50 mM MgCl2, 200 μM primer (forward and reverse) and appropriate volume of sterile water. Primer sequences and combinations for *TSC1* were used as described elsewhere in detail [22]. The parameter for amplification of *TSC1* was predenaturating at 95°C for 5 min followed by 35 cycles at 95°C for 1 min, annealing at 55°C for 1 min and 72°C for 1 min and a final extension at 72°C for 7 min in an automated thermocycler (MJ Research PTC-200 Thermo Cycler). After PCR amplification 2 μl of each product supplemented with loading buffer (Invitrogen) and a marker (100 bp Molecular Ruler, BIO-RAD) were electrophoresed in 2.5% agarose gel, stained with ethidium bromide (Invitrogen) and then photographed under ultraviolet (UV) light. The comparison of marker and size of the amplification product ensured the presence of the wanted DNA section.

For DNA sequencing, excess primers and residuals were removed from the remaining PCR Product by PEG precipitation as described elsewhere in detail [23] and PCR products were dissolved in a final volume of 20 μl. DNA was quantified by measuring the UV absorption and the quality was examined by electrophoresis. The extended fragments were sequenced with the BigDye® Terminator v1.1 Cycle Sequencing Kit (applied Biosystems, Germany) according to the manufacturer’s instructions in the ABI PRISM® 310 Genetic Analyzer (applied Biosystems, Germany) and analysed by comparison with the GenBank sequence file.

### Statistical analyses

Expression data of both whole block sections and TMA sections were subjected to statistical analyses. Associations between immunohistochemical expression, the clinical follow up and preliminary data concerning EGFR mutations were tested by Pearson’s 2-sided *Χ*^2^ test. Correlation coefficients for immunohistochemical correlations were estimated by Kendall's rank correlation. All cutoff values of significance were set p < 0.05 with 2-sided testing. Survival proportions were assessed using the Kaplan-Meier method (IBM SPSS Statistics 20).

## Results

### Inverse correlation of hamartin and p-mTOR expression in human lung cancer cell lines

**Figure 2:**
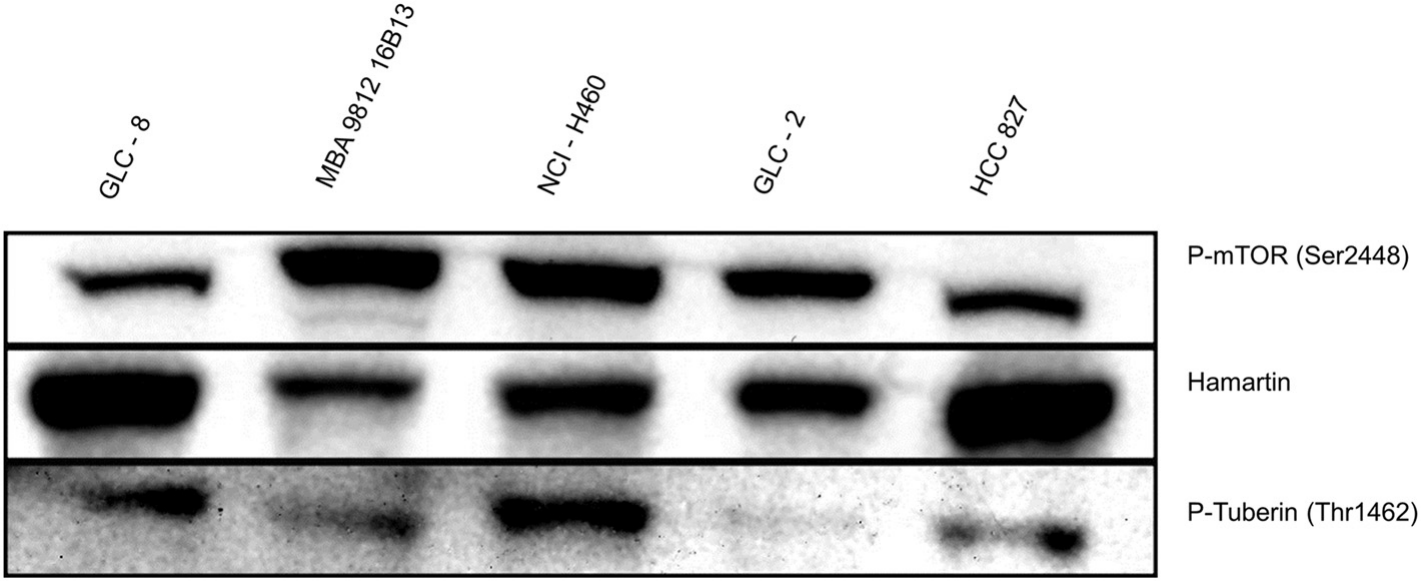
**Western blot analysis for p-mTOR, TSC1 and p-tuberin (Thr1462) in NSCLC and SCLC cell lines.** In GLC-8, MBA 9812 and HCC-827 cells, we found an inverse correlation between hamartin and p-mTOR concentrations. Higher TSC1 levels were associated with low levels of p-mTOR and, vice versa a substantial expression of p-mTOR was obtained in association with decreased levels of TSC1. There was no obvious correlation between p-tuberin levels with those of hamartin or p-mTOR. *NCI-H460:* large cell cancer of the lung, *MBA 9812:* SCC, *HCC-827:* AC harboring an acquired mutation in the EGFR tyrosine kinase domain (E746 - A750 deletion), *GLC-2* and *GLC-8:* SCLC.

We performed western blot analyses using cultured cells of SCLC (cell lines: GLC-2 and GLC-8) and NSCLC (cell lines: NCI-H460, MBA 9812, HCC-827). We found hamartin, p-tuberin and p-mTOR protein expression by western blot in all cell lines. GLC-8, MBA 9812 and HCC-827 cells showed an inverse correlation between hamartin and p-mTOR expression. Higher hamartin levels were associated with low levels of p-mTOR and, vice versa a substantial expression of p-mTOR was obtained in association with decreased levels of hamartin. No correlation between p-tuberin and hamartin or p-mTOR was found (Figure 2).

### Immunohistochemical analysis of hamartin, p-tuberin and p-mTOR: hamartin is expressed in a large proportion of cases

**Figure 3:**
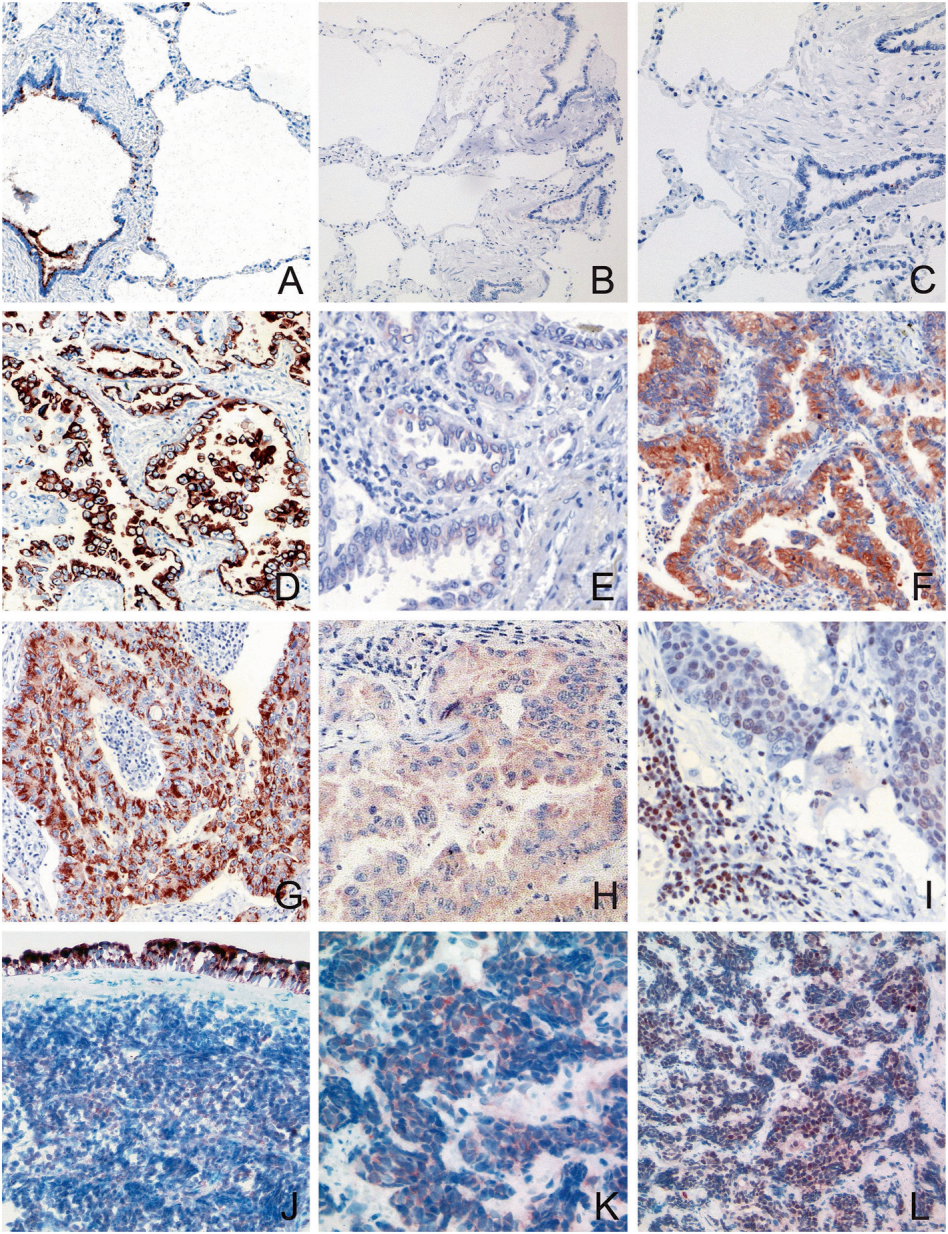
**Immunohistochemistry.** In normal tissue, only TSC1 is expressed in bronchus epithelia but not in alveolar surface cells **(A)**. P-mTOR and p-tuberin were not detected in normal tissue **(B, C)**. TSC1 was expressed in more than 50% of AC **(D)** and SCC **(G)** in contrast to 14% of SCLC **(J)**. P-TSC2 was weakly expressed in 16.3% of AC **(E)**, 6.5% of SCC **(H)** and 4.7% of SCLC **(K)**. P-mTOR expression was found either in the cytoplasm or in nuclear position **(F, I, L)**. Nuclear p-mTOR staining was found in 22.8% of AC **(F)**, 35.5% of SCC **(I)** and 16.3% of SCLC **(L)**. Cytoplasmic labeling of p-mTOR was found in 21.8% of AC **(F)** and 9.7% of SCC **(I)**, but not in SCLC **(L)** specimens. *Figures A-C: normal lung, D-F: AC, G-I: SCC, J-L: SCLC.*

Cytoplasmic hamartin staining was found in a substantial proportion of AC revealing a strong cytoplasmic expression in 40.2% and a moderate expression in additional 18.5%. SCC also revealed hamartin in slightly more than 50% (strong expression in 29% and moderate expression in 25.8%). In contrast, only 14% of SCLC expressed hamartin (strong expression in 9.3% and moderate expression in another 4.7%). P-mTOR expression was found either in the cytoplasm or in nuclear position. Nuclear staining was found in 22.8% of AC (strong labeling in 4.3% and moderate staining in 18.5%) and in 35.5% of SCC (strong expression in 6.5% and moderate expression in 29%). SCLC less frequently expressed p-mTOR in the nucleus (4.7% strong / 11.6% moderate). Cytoplasmic labeling of p-mTOR was found in 21.8% of AC (strong labeling in 3.3% and moderate staining in 18.5%), 9,7% in SCC (0% strong / 9.7% moderate expression), but was not detected in SCLC specimens. Cytoplasmic p-TSC2 expression was found in 16.3% of AC (moderate to strong staining) compared to moderate expression in 6.5% of SCC resp. 4.7% of SCLC (Figure 3).

In non-neoplastic control tissue, hamartin was expressed in bronchus epithelia with accumulation in the apical submembranous compartments of the cells. It was not detectable in the alveolar epithelial cells. P-mTOR and p-tuberin were not detected in bronchiolar or alveolar epithelial cells immunohistochemical (Figure 3).

Furthermore, we screened for correlations between hamartin, p-mTOR and p-TSC2. A significant correlation between the expression of p-TSC2 and p-mTOR was found in AC specimens (p-TSC2 vs. nuclear p-mTOR: correlation coefficient (CC) = 0.305; p = 0.004; p-TSC2 vs. cytoplasmic p-mTOR: CC = 0.303; p = 0.016) and in SCLC specimens (p-TSC2 vs. nuclear p-mTOR: CC = 0.409; p = 0.006; pTSC2 vs. cytoplasmic p-mTOR: CC = 0.422; p = 0.005). In SCLC, the expression of hamartin correlated with that of p-TSC2 (CC = 0.669; p < 0.001) and with the expression of nuclear p-mTOR (CC = 0.405; p = 0.007). In SCC patients no significant correlations were revealed.

### Immunohistochemical correlation with signaling pathways upstream of TSC-mTOR reveals a substantial co-expression of hamartin and phosphorylated EGFR at position Tyr-1068 as well as with p-EGFR at position Tyr-992

We correlated the expression of hamartin, p-TSC2 and p-mTOR with expression data concerning epidermal growth factor receptor mutations in non-small cell lung cancer and their influence on downstream Akt, MAPK and Stat3 signaling [21]. In AC specimens, we found a substantial co-expression of hamartin and phosphorylated EGFR at position Tyr-1068 (CC = 0.472; p = 0.001) as well as with p-EGFR at position Tyr-992 (CC = 0.706; p < 0.001). Phosphorylation of mTOR was closely correlated with p-EGFR Tyr-1173 (CC = 0.643; p < 0.001) (Table 1). In SCC specimens, an inverse correlation was found between hamartin and p-EGFR Tyr-992 (CC = −0.688; p = 0.001). Furthermore, p-TSC2 was inversely correlated with expression of MAP-Kinase (CC = −0.708; p = 0.003).

**Table 1:** Correlation of hamartin and p-mTOR with expression data concerning phosphorylated epidermal growth factor receptor in adenocarcinoma

**EGFR mutation**	**Test**	**Hamartin**	**P-mTOR**
Phospho-EGFR Tyr-992	Pearson correlation	- 0,052	0,404^b^
	Sig. (2-tailed)	0,728	0,027
	N	47	30
Phospho-EGFR Tyr-1173	Pearson correlation	0,027	0,643^a^
	Sig. (2-tailed)	0,859	< 0,001
	N	46	30

^a^Correlation is significant at the 0.01 level (2-tailed).

^b^Correlation is significant at the 0.05 level (2-tailed).

### Mutation analyses in NSCLC and SCLC cells showed one sequence alteration in exon 23

We considered whether accumulation of hamartin was due to *TSC1* sequence alterations. Sequence alterations of *TSC1* were found in only one cell line, i.e. the “HCC827” cells, i.e. an adenocarcinoma cell line harboring an acquired mutation within the EGFR tyrosine kinase domain. We only detected one sequence alteration in exon 23 with multiple base pair transitions leading to a complex alteration of the amino acid composition, but without truncation of the protein.

### P-mTOR expression tended to be more frequently among EGFR + cases when correlating immunohistochemical data with EGFR mutation status

We then wondered whether expression of hamartin, p-mTOR or p-TSC2 was associated with EGFR-mutations since approximately 20% of adenocarcinoma of the lung patients’ specimens reveal EGFR-mutations [24]. To address this issue, we analysed 12 cases with established EGFR mutation status for therapeutic purposes (each 6 cases with/without EGFR mutations) and evaluated the tumor specimens for hamartin, p-tuberin and p-mTOR expression. For hamartin 2 of 6 tumors harboring EGFR mutations (EGFR+) showed an expression level >2, compared to 1 case in the group of EGFR wild-type tumors (EGFR-) showing an expression level >2. For p-tuberin, in each group (EGFR+ / EGFR-) only one case showed an expression level 1. Four of six EGFR + tumors weakly expressed p-mTOR compared to two of six cases without mutated EGFR. P-mTOR expression tended to be more frequently among EGFR + cases. Concerning hamartin and p-tuberin, there was no obvious association to the EGFR mutation status (Table 2).

**Table 2:** Staining of AC with established EGFR mutation status

	**EGFR sequence alterations**			**Immunohistochemical analysis**		
**No.**	**EGFR mutation**	**Mutation**	**Exon**	**TSC1**	**P-TSC2**	**P-mTOR**
1	Yes	c.2573 T > G; p.L858R	21	---	---	---
2	Yes	p.E746_P753 delinsIS c.2236_2259(24 bp)delinsATCTCG	19	+	+	+
3	Yes	c.2236_2250del p.E746_A750del	19	---	---	---
4	Yes	p.L858R c.2573 T > G	21	---	---	+
5	Yes	c.2235_2249del p.E746_A750del	19	---	---	+
6	Yes	p.L858R	21	+	---	+
7	No	Wild type		---	---	---
8	No	Wild type		+	---	---
9	No	Wild type		---	---	---
10	No	Wild type		---	---	+
11	No	Wild type		---	+	+
12	No	Wild type		---	---	---

### The clinical follow-up shows that hamartin staining is associated with poorer survival in a subset of lung cancer entities

**Figure 4:**
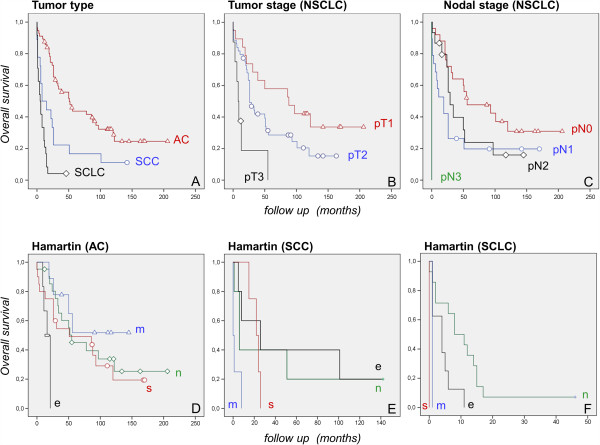
**Kaplan-Meier curves for different tumor types.** The clinical course (overall survival) (OS) was dependant on the tumor type (AC vs. SCC vs. SCLC) **(A)**, the extent of the tumor (pT) **(B)** as well as the extent of lymphonodular spread (pN) **(C)**, indicating a representative tumor cohort in the present study. In SCC and SCLC specimens, moderate resp. strong hamartin expression survival was significantly poorer compared to cases with no expression or equivocal staining characteristics (p = 0.014 resp. p < 0.01) **(E, F)**. This effect was not obtained for AC specimens (p > 0.05) **(D)**. *n: negative, e: equivocal, m: moderate, s: strong hamartin expression.*

Overall, follow up data were available in 98 of 166 cases (59.0%). 76 of 98 patients died within the follow up period (77.5%). The remaining 22 patients were documented as ’still alive’ at the last available time point. Only in six patients without documentation of death the follow-up period was less than 60 months. The median survival of AC cases was 51 months (SD 9.7; 95% CI: 31.9 – 70.0; n = 56), compared to SCC with 15 months (SD 8.2; 95% CI: 0 – 31.1; n = 17), and SCLC with 5 months (SD 1.9; 95% CI: 1.2 – 8.8; n = 24). As expected, the histological tumor type (AC vs. SCC vs. SCLC) influenced the overall survival characteristics, in accordance with published clinicopathological data for lung cancer [1,25]. It should be noted that AC and SCC patients included in the present study were treated with a curative aim, whereas the collective of SCLC patients represent cases with both curative and palliative regimens. Moreover, survival was dependant on the extent of the tumor (pT) as well as the lymphonodular spread (pN) (Figure 4).

Based on the four-tiered semiquantitative expression analysis, immunohistochemical hamartin staining was significantly associated with a poorer survival in SCC and SCLC patients (Figure 4). In SCC specimens with moderate resp. strong hamartin expression survival was significantly poorer (mean survival 2.25 resp. 21.75 months) compared to cases with no expression or equivocal staining characteristics (mean survival 50.0 resp. 56.4 months) (p = 0.014). The same negative prognostic effect was found in SCLC cases (mean survival of 0.5 resp. 1 month in SCLC specimens with moderate resp. strong hamartin expression vs. 11.2 resp. 4.1 months in cases with no expression or equivocal staining) (p < 0.01). For AC specimens the Kaplan-Maier plot revealed no effect of hamartin expression on survival (mean survival of 93.9 resp. 71.9 months for moderate resp. strong hamartin expression vs. 90.3 resp. 16.5 months in cases with no expression or equivocal staining) (Figure 4).

Expression of cytoplasmic or nuclear p-mTOR as well as expression of p-TSC2 did not influence survival proportions (all p > 0.05).

## Discussion

In the present study we examined the morphological characteristics and functional role of the TSC tumor suppressor complex as a major regulator of mTOR activity. We could show that the TSC tumor suppressor complex is substantially expressed in lung cancer cell lines and that hamartin and p-mTOR were inversely correlated in three of the five lung cancer cell lines. Furthermore, slightly more than 50% of the NSCLC specimens showed hamartin expression, compared to nearly one third of SCLC with valuable hamartin expression. These findings demonstrate that hamartin expression is a frequent finding in lung cancer. A major challenge is to assess whether accumulation or loss of hamartin (and p-tuberin) reflects primary or secondary events. Both, accumulation or loss of hamartin, could be pathogenically relevant for carcinogenesis. As in normal tissue hamartin is only expressed in bronchial respiratory epithelia but not in alveolar epithelial cells, we cannot conclude if hamartin expression reflects a gain or loss of function in tumor specimens. In NSCLC and SCLC cell lines, high protein levels of hamartin were associated with low p-mTOR and vice versa. This inverse correlation between hamartin and mTOR levels supports an interaction between TSC and mTOR in NSCLC and SCLC. All cell lines used for the present study revealed detectable hamartin and p-TSC2 protein levels indicating that expression differences are rather due to a loss of hamartin expression. This interpretation is also reasonable in the light of previous studies showing a loss of heterozygosity (LOH) of the TSC1-locus on chromosome 9q34 in AC and precursor lesions [18]. Another study also reported LOH for hamartin or TSC2 in 22% of 86 specimens, but none of the 80 lung cancer lines studied [20].

In SCLC, we found that hamartin expression correlates with p-TSC2 and may point towards a disruption of the hamartin-tuberin complex, which is accompanied by phosphorylation of tuberin and activation (i.e. phosphorylation) of mTOR. Moreover, the expression of hamartin correlated with that of nuclear p-mTOR (CC = 0.405; p = 0.007) suggesting that hamartin may be an interesting surrogate marker for mTOR related signaling. The immunohistochemical characterization of signaling pathways during the routine histological workup of specimens would greatly facilitate the selection of individualized therapeutic regimens that are currently arising by the availability of new molecular targets such as mTOR inhibitors [26].

Growing evidence supports abnormally activated mTOR to play an important pathogenic role in lung cancer associated with both KRAS and EGFR mutations and may provide a mechanism of resistance to treatment with EGFR inhibitors [26,27]. The EGFR can be autophosphorylated on various tyrosine sites and distinct downstream signaling cascades are initiated by the EGFR depending on its phosphorylation pattern [28,29]. As EGFR signaling is partially mediated via KRAS and both KRAS and EGFR can activate PI3K, a potential link with TSC is reasonable (Figure 1). A potential interaction between TSC and KRAS has been postulated in mice [20]. Tumors of animals harboring hamartin loss and KRAS (G12D) expression in lung epithelial cells revealed 1) reduced tumor latency, 2) an activation of mTOR and 3) a response to treatment with rapamycin with improved survival compared to KRAS alone mutant mice [20]. These observations suggest that the TSC complex may be a critical regulator of KRAS-related signaling cascades that are focusing on mTOR. Overall, these data support a rather complex, interdependent regulation of the TSC complex and the EGFR/KRAS signaling. We have therefore used immunohistochemical data of a recent study and speculated if abnormal activation of mTOR is due to pathogenic events upstream of the hamartin-tuberin-complex. Based on this approach, we found a significant correlation between hamartin and p-EGFR (Tyr-1068) resp. p-EGFR (Tyr-992) expression in AC specimens [21]. P-mTOR was also closely correlated with p-EGFR Tyr-1173. These findings indicate that expression or even accumulation of hamartin may also be secondary to EGFR phosphorylation. In contrast, an inverse correlation was found between hamartin and p-EGFR Tyr-992 in SCC specimens indicating different molecular fingerprints in different cancer subtypes.

We have also hypothesized that hamartin, p-tuberin and p-mTOR expression may be dependant of the EGFR mutation status. In 12 cases with already established EGFR mutation status for therapeutic purposes, hamartin accumulation was found both in EGFR-mutated and EGFR wild type tumors (Table 2). Also p-tuberin expression was detected both in EGFR + and EGFR- cases. Nuclear expression of p-mTOR was slightly more frequent in patients harboring EGFR mutations, however it was also detectable in EGFR-wild type cases. Therefore, we conclude that the EGFR mutation status does not affect expression of hamartin, p-tuberin and p-mTOR responsive to EGFR mutations. This assumption is also supported by the observation that phosphorylation of tyrosine residues 922 and 1173, but not phosphorylation of tyrosine residues 1068, have been associated with activating EGFR mutations [21].

We have also raised the question, if accumulation of hamartin may be secondary to mutational alterations. Notably, LOH for the *TSC1* or *TSC2* locus has been described in 22% of 86 human lung cancer specimens [20]. In another study more than one third of atypical adenomatous hyperplasia precursor lesions and 53% of concomitant adenocarcinomas displayed LOH on 9q. A substantial proportion of these harbored LOH at loci adjacent to the TSC1 gene [18]. However, only one cell lines used in the present study revealed a *TSC1* sequence alteration, i.e. the HCC-827 cell line (AC harboring an acquired mutation in the EGFR tyrosine kinase domain). This sequence alteration was represented by complex alterations in the close vicinity of the stop codon in exon 23 of *TSC1*. We suggest that this alteration is not functionally relevant as it is located beyond the critical tuberin interaction domain and as it does not result in a truncation of the protein.

Regardless of its possible pathogenic role, hamartin expression may provide as a prognostic marker. Clinical follow up data were available in nearly two third of the cases. As expected the histological tumor type strongly influenced the survival rates. The longest mean survival was observed in AC patients and the shortest in SCLC patients. Regarding TSC-related expression, SCC and SCLC patients revealed a poorer overall survival in hamartin positive cases. In contrast, no prognostic effect of hamartin expression could be observed for AC specimens. Differences regarding its prognostic value may also reflect different molecular pathways involved during carcinogenesis resp. therapeutic strategies. Nevertheless, other independent factors with a potential influence on survival that have recently been discussed could be considered, e.g. an overexpression of MTA3 gene in NSCLC as a risk factor on survival [30] or an overexpression of IMP3 as a predictor of aggressive tumor behavior [31]. Further investigations in this direction should follow.

In contrast, we could not reveal a prognostic influence of p-tuberin and p-mTOR. In another study focusing on NSCLC, positive cytoplasmic mTOR staining was associated with shorter survival [32]. Furthermore, high mTOR expression has been claimed to be associated with a worse outcome in laryngeal squamous cell carcinomas that have been subjected to postoperative radiotherapy [33]. The discrepancy between our results and the aforementioned studies may be due to the fact, that we recruited an antibody directed only against phosphorylated (i.e. activated) mTOR, implicating a limited comparability of these results. Furthermore, we have observed p-mTOR expression both in the cytoplasm and nucleus and the functional relevance of nuclear mTOR has yet to be elucidated. Classically, mTOR acts in the cytoplasm, but recent findings have supported compartment-specific mTOR functions in other subcellular compartments including the nucleus [34]. The existence of a nuclear shuttling signal in mTOR has been postulated being essential for nuclear mTOR import [35]. These findings fit well with our observations that p-mTOR was detected not only in the cytoplasm but also in the nucleus of tumor cells in immunohistochemical observations. Nuclear p-mTOR staining was found in 22.8% of AC, 35.5% of SCC and 16.3% of SCLC specimens. Thus, the different staining frequencies in the different tumor types may also reflect the compartment-dependant diversity of mTOR signaling. P-mTOR has also been assigned to aggressive histological variants of papillary thyroid carcinoma and, thus nuclear labeling of p-mTOR has been discussed to serve as a diagnostic and prognostic marker as well as a potential therapeutic target [36].

## Conclusions

In Summary, our results indicate hamartin, p-tuberin and p-mTOR expression in a substantial subset of NSCLC and SCLC specimens. We hypothesize a relevant regulation of mTOR by the TSC complex in NSCLC and SCLC cells. Our results support TSC activation corresponding to EGFR and MAPK signaling, but there is no established association with distinct EGFR mutations. Finally, our findings support hamartin expression as a negative prognostic marker in SCC and SCLC cases.

## Competing interests

The authors declare that they have no competing interests.

## Authors’ contributions

AF conducted and evaluated experiments (Western blot, cell lines), managed the clinicopathological correlation (follow-up) and drafted the manuscript. KK and LCH instructed scientific work and assisted to conduct the experiments (Western blot, cell culture systems, TMA creation). JF and JK implemented molecular investigations for EGFR mutation status. SH drawed up clinicopathological correlation (follow-up) and statistical evaluations. AJB provided reagents and assistance regarding mutation analysis of TSC1 in the cell lines. RB head of our research institution provided human and material resources. MM performed immunohistochemical assessment and evaluation of histological preparations, clinicopathological correlation (follow-up), including database setup and statistical analysis, helped to draft the manuscript. All authors read and approved the final manuscript.
